# Unmet need in pulmonary hypertension-associated interstitial lung disease (PH-ILD): a clinician survey of real-world management of PH-ILD in Europe

**DOI:** 10.1183/23120541.00039-2024

**Published:** 2024-07-08

**Authors:** David Montani, José M. Cifrián, Raquel P. Rojo, Hilario Nunes, Federica Meloni, Stefano Ghio, John Cannon, Andreas Günther, Héctor Gálvez García, Míriam Fernández Delgado, Gabriela Silvina Bacchini Jeanneret, Luke Howard

**Affiliations:** 1 Université Paris–Saclay, AP-HP, INSERM UMR_S 999, Department of Respiratory and Intensive Care Medicine, Pulmonary Hypertension National Referral Centre, Hôpital de Bicêtre, Le Kremlin Bicêtre, France; 2 Hospital Universitario Marqués De Valdecilla, Pneumology service, Santander, Spain; 3 12 de Octubre Hospital, Pneumology Department, Madrid, Spain; 4 Hôpital Avicenne, AP-HP, Service de Pneumologie, Centre de référence des maladies pulmonaires rares, Université Sorbonne Paris Nord, Bobigny, France; 5 Fondazione Policlinico San Matteo and University of Pavia, UOS Transplant Center, Pavia, Italy; 6 Fondazione IRCCS Policlinico S.Matteo, Divisione di Cardiologia, Pavia, Italy; 7 Royal Papworth Hospital, Cambridge, UK; 8 Center for Interstitial and Rare Lung Diseases, Justus-Liebig-University Giessen, Giessen, Germany; 9 Ferrer, Corporate Medical Affairs, Barcelona, Spain; 10 Imperial College Healthcare NHS Trust, London, UK

## Abstract

**Background:**

With no approved therapies for pulmonary hypertension (PH) associated with interstitial lung disease (PH-ILD) in Europe, we surveyed clinician perceptions on PH-ILD management and unmet need to understand current real-world practices.

**Methods:**

An online clinician survey on PH-ILD management was conducted in France, Germany, Italy, Spain and the UK.

**Results:**

55 clinicians (78% pulmonologists), each managing a median 20 PH-ILD patients (interquartile range (IQR) 10–50), participated. Upon PH suspicion, clinicians referred a median 50% (IQR 20–73%) of patients for echocardiography alone and 35% (IQR 20–78%) for echocardiography, followed by right heart catheterisation. Upon diagnosis, a median 20% (IQR 9–30%), 40% (IQR 20–50%) and 35% (IQR 20–55%) of patients fell under the pulmonary arterial pressure ranges of 21–24 mmHg, 25–34 mmHg and >35 mmHg, respectively. 50% of patients received off-label treatment for their PH and, of those, off-label phosphodiesterase-5 inhibitor (PDE-5i), endothelin receptor antagonist (ERA) and prostacyclin analogues were prescribed first-line by 78%, 9% and 7% of clinicians, respectively. Upon PDE-5i non-response, 35% of clinicians proceed with an ERA, 35% with no further therapy. 55% of clinicians used dual-therapy. Yearly median inpatient admissions and emergency visits were 2.0 (IQR 1.3–2.9) and 1.5 (IQR 1.0–2.0), respectively (n=31 responses). Most clinicians (69%) highlighted lack of efficacy or evidence for current therapies as a key gap in PH-ILD management.

**Conclusions:**

This study gives insight into real-world European PH-ILD diagnosis and management. With significant use of off-label treatment, there is a large unmet need due to lack of approved therapies. Despite updated guidelines, more evidence is needed to standardise PH-ILD management.

## Shareable abstract

**Only around half of ILD patients suspected to have PH are diagnosed, and of these, only half are receiving treatment, albeit off-label, for their PH. There is a large unmet need for efficacious PH-ILD therapies for this severe disease.** https://bit.ly/3wGx09F

## Introduction

Interstitial lung diseases (ILD) are rare and often progressive lung interstitium-based disorders. ILD patients can develop pulmonary hypertension (PH), a common complication characterised by elevated mean pulmonary arterial pressure (mPAP). This condition, known as PH associated with ILD (PH-ILD), is associated with a higher risk of mortality, acute exacerbation, poor quality of life (QoL) and increased need for supplemental oxygen therapy compared to ILD alone [1, 2].

The European Society of Cardiology (ESC) and European Respiratory Society (ERS) 2022 guidelines classify PH as an mPAP of >20 mmHg and pulmonary vascular resistance (PVR) of > 2 Wood units confirmed by right heart catheterisation (RHC) [1]. Within the ESC/ERS 2022 guidelines, PH is divided into five groups, with PH associated with lung disease or hypoxia classified as group 3. Accordingly, PH-ILD falls under group 3 PH. Severe group 3 PH is defined in the guidelines to be a PVR of >5 Wood units. The prevalence of group 3 PH varies, with most epidemiology studies conducted in individual countries. The estimated prevalence of group 3 PH and PH-ILD is around 4 per 10 000 [2] and between 0.8 and 1 per 10 000 respectively [3–5].

Group 3 PH has the lowest survival out of the five groups. A longitudinal UK PH cohort showed the shortest median survival for group 3 PH (around 21 months) [6] compared to other PH groups. The presence of PH of any severity has been shown to negatively impact survival in both COPD [7], a subcategory of group 3 PH, and in ILD [8]. However, within group 3 PH, PH-ILD is the most severe subgroup for mortality, as the 3-year survival rate was shown to be better for PH-COPD than for PH-ILD (57% *versus* 33% for those with severe PH) [8]. Group 3 PH and, by extension, PH-ILD, therefore are very severe diseases that are unique amongst the PH groups in their clinical burden.

Pulmonary arterial hypertension (PAH, group 1 PH)-approved therapies target pathways involved in endothelial dysfunction, thereby reducing PVR through triggering vasodilation and reducing PH. Phosphodiesterase 5 inhibitor (PDE-5i) and endothelin receptor antagonist (ERA) therapies inhibit PDE-5 or compete for the ER, respectively, whereas soluble guanyl cyclase (sGC) stimulator and prostacyclin analogue (PCA) therapies promote the production of vasodilatory intermediary messengers. Except for inhaled treprostinil (INCREASE study; [9]), no PAH therapies have met their primary end-point in PH-ILD trials [1]. Therefore, there are no approved targeted treatments for PH-ILD in Europe. Some PH-ILD patients are managed with off-label PAH therapies; however, these medications have limited and conflicting evidence in PH-ILD and can cause adverse events affecting gas exchange and haemodynamics [1]. Managing PH-ILD requires consideration of the interaction between PH and ILD, as different approaches impact either the PH or ILD component. Standardising PH-ILD treatment is challenging due to underlying ILD diversity. In 2021, the US Food and Drug Administration (FDA) approved inhaled treprostinil, a PCA, for treating PH-ILD. To date, inhaled treprostinil has not yet received approval in Europe.

Recommendations from the 2022 ESC/ERS guidelines suggest confirming PH by RHC in ILD where lung transplant and therapeutic interventions are considered [1]. Once diagnosed, recommended treatment includes supplemental oxygen therapy and enrolment into pulmonary rehabilitation programmes. The only reference to PH management specifically is that “inhaled treprostinil may be considered” based on the INCREASE study [9] with more information being needed and that severe PH warrants referral to a PH centre for “individualised decision making”. Optimal PH-ILD patient management is still being explored, for example with the recent publication of a diagnostic Delphi panel in the US [10], but many knowledge gaps remain in patient management [11].

Owing to the lack of evidence-based guidance on PH-ILD diagnosis and treatment, an online clinician survey was conducted to investigate current PH-ILD management practices and clinical outcomes in PH-ILD patients across France, Germany, Italy, Spain and the UK.

## Methods

Pulmonologists, cardiologists and rheumatologists in France, Germany, Italy, Spain and the UK were identified in literature searches or listings of pulmonology services throughout Europe and invited to take part in an online questionnaire-based survey about their perceptions on the unmet need in PH-ILD management in Europe. Participants who consented and confirmed their expertise as either cardiology, pulmonology or rheumatology in screening questions and who managed PH-ILD patients were selected. All personal details were processed in accordance with general data protection regulation.

The questionnaire comprised 73 questions in the English language covering clinician characteristics, patient characteristics, treatment pathway and patient outcomes (see supplementary material item 1 for full questionnaire). The questionnaire was hosted on the SmartSurvey platform (www.smartsurvey.co.uk/). Responses were collected between August 2022 and October 2022. Respondents were given a voluntary hospitalisation rate follow-up question in December 2022. Descriptive analysis was carried out on non-free text questions, with data reported as median values with interquartile ranges (IQR) from the 25th to the 75th percentile. Data were tested for normalcy using the Shapiro–Wilk test. For categorical variables, results were expressed as percentages.

## Results

### Clinician characteristics

55 clinicians completed the survey, including 12 clinicians who practised in France, 11 in Spain, Italy and the UK respectively, and 10 in Germany. Over three-quarters were pulmonologists (78%), more than half managed both PH and ILD (62%) (the remainder managed only PH (16%) or ILD (22%)) and about three-quarters practised at academic or research-based healthcare centres (73%). Most clinicians had echocardiography (100%) and RHC (95%) capabilities and over half had lung transplant (51%) capabilities at their centres. Clinicians managed a median of 110 ILD (n=44 responses, IQR 50–200), 73 PH (n=45 responses, IQR 20–185) and 20 PH-ILD patients (n=50 responses, IQR 10–50) annually. Nearly three-quarters (71%) of the respondents had >10 years of experience managing PH/ILD/PH-ILD. A similar proportion participated in relevant PH/ILD/PH-ILD registries (71%) and 65% were involved in clinical trials.

### Patient characteristics

Over half of patients were reported to be male (median 60%, IQR 50–70%), nearly three-quarters had World Health Organisation (WHO) functional class III or IV (median 80%, IQR 61–89%). The main causes of ILD were idiopathic pulmonary fibrosis (IPF) (median 25%, IQR 15–35%), scleroderma-associated ILD (median 22%, IQR 15–38%) and combined pulmonary fibrosis and emphysema (median 12%, IQR 9–20%) (table 1). Dyspnoea, fatigue and cough were the symptoms most reported to be highly frequent (present in >50% of their patients) by the clinicians (100%, 80% and 58% of clinicians, respectively).

**TABLE 1:** Reported characteristics of PH-ILD patients personally managed by clinicians (n=55)

Parameter		
	Mean±sd %	Median (Q1–Q3)
**Gender**		
** **Male	58±16	60 (50–70)
** **Female	42±16	40 (30–50)
**Age**		
** **≥60 years old	73±14	75 (63–80)
** **<60 years old	27±14	25 (20–38)
**Time since ILD diagnosis (n*=*52)**		
** **≥2 years	68±20	70 (58–81)
** **<2 years	32±20	30 (19–43)
**Time since PH diagnosis (n*=*51)**		
** **<6 months	32±28	25 (10–30)
** **6–12 months	28±16	30 (20–40)
** **>12 months	41±24	40 (30–60)
**ILD type or cause**		
** **IPF	29±19	25 (15–35)
** **NSIP	11±8	10 (5–15)
** **Scleroderma-CTD	30±23	22 (15–38)
** **Non-scleroderma-CTD	9±7	10 (5–10)
** **Sarcoidosis	9±8	5 (4–10)
** **CPFE	15±12	12 (9–20)
** **Others^#^	4±5	5 (0–8)
**WHO functional class (n*=*54)**		
** **I–II	28±21	20 (11–39)
** **III–IV	72±21	80 (61–89)
**Forced vital capacity**		
** **<50% predicted	33±19	30 (20–40)
** **50–70% predicted	46±18	45 (30–60)
** **>70% predicted	22±16	20 (13–30)

IPF: idiopathic pulmonary fibrosis; PH-ILD: pulmonary hypertension associated with interstitial lung disease; Q1–Q3: quartile 1 to quartile 3; ILD: interstitial lung disease; PH: pulmonary hypertension; NSIP: nonspecific interstitial pneumonia; CTD: connective tissue disease; CPFE: combined pulmonary fibrosis and emphysema; WHO: World Health Organisation. ^#^: *e.g.*, pulmonary amyloidosis, chronic hypersensitivity pneumonitis.

### Diagnosis

The diagnostic algorithms that were referenced for diagnosing PH were the ones outlined in the 6th World Symposium on Pulmonary Hypertension (WSPH) and the ESC/ERS 2022 guidelines. 44% of clinicians adhered to the 6th WSPH guidelines, while 53% followed the ESC/ERS guidelines for diagnosing PH. The remainder (n=2) indicated that they did not know which guidelines were used to diagnose PH.

Regarding the exams used to suspect PH, clinicians selected from a list of potential signs: abnormal pulmonary function test (PFT) (low diffusing capacity of the lung for carbon monoxide (*D*_LCO_) and elevated % forced vital capacity (FVC)/% *D*_LCO_ ratio) (82%), high mPAP (78%) and specific imaging findings (67%), such as enlarged pulmonary artery. Upon PH suspicion, 49 clinicians (89%) referred their ILD patients to an in-house PH specialist for a RHC to confirm the diagnosis of PH, whereas 11% of clinicians referred their ILD patients to an out-house PH specialist.

**FIGURE 1:**
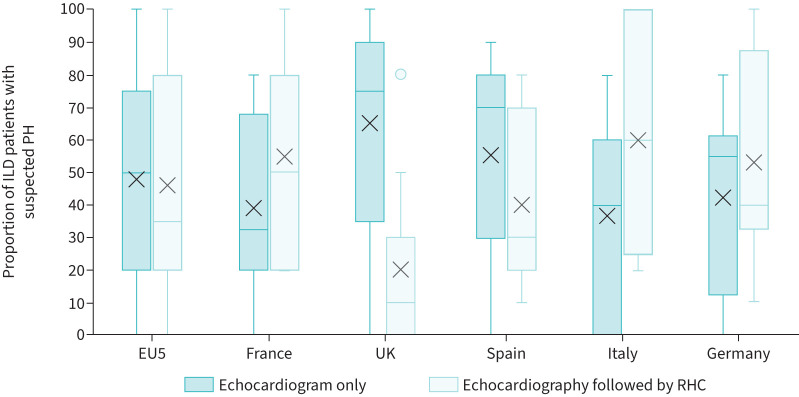
Diagnostic procedures carried out by clinicians to confirm PH in ILD patients suspected to have PH (n=55). EU5: European five countries (France, Germany, Italy, Spain and the UK); ILD: interstitial lung disease; PH: pulmonary hypertension; RHC: right heart catheterisation.

Work up on suspected PH was based on echocardiography alone and RHC after echocardiography in a median 50% (IQR 20–73%) and 35% (IQR 20–78%) of PH-suspected ILD patients respectively (figure 1). Median RHC usage to confirm PH in suspected ILD patients varied among countries: Italy (60%, IQR 30–93%), France (50%, IQR 30–80%), Germany (40%, IQR 35–81%), Spain (30%, IQR 25–60%) and the UK (10%, IQR 5–25%).

Current guidelines do not explicitly delineate what constitutes sufficient suspicion to carry out RHC in suspected ILD patients. Thus, clinicians provided further insights by free-text answers into their decision-making process regarding RHC. The largest portion (24%) explained that RHC is carried out if the ILD patient is an ideal candidate to receive off-label PAH treatment or eligible for lung transplantation. Additionally, 16% answered that RHC would be performed in mild-to-moderate ILD patients with moderate-to-severe PH or when echocardiogram indicates high PH probability (supplementary table S1). Clinicians stated that RHC was not used in severe ILD (15% of clinicians) or if the procedure would not be tolerated (13% of clinicians). With regards to severity of PH at diagnosis, a median 20% (IQR 9–30%) of PH-ILD patients had 21–24 mmHg, 40% (IQR 20–50%) had 25–34 mmHg and 35% (IQR 20–55%) had pressure above 35 mmHg (n=52, three clinicians did not have this information).

For perceptions on possible improvements to the diagnostic pathway, clinicians recommended more frequent echocardiogram screening in ILD (35% of clinicians) and increased PH disease awareness and education amongst non-PH specialists (24%). Clinicians also highlighted the need for more effective screening tools (15%) and rapid and easy access to RHC (13%) (supplementary table S1).

### Treatment

**FIGURE 2:**
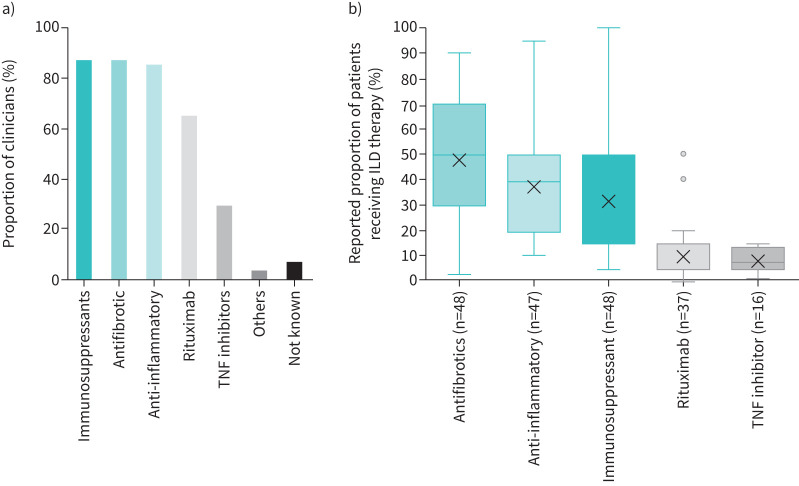
Distribution of a) clinician prescription of ILD drugs in PH-ILD patients and b) clinician-reported PH-ILD patient receipt of ILD therapies (n=16–55^#^). “Others” consists of cyclophosphamide and immunoglobulins. ILD: interstitial lung disease; PH-ILD: pulmonary hypertension associated with interstitial lung disease; TNF: tumour necrosis factor. ^#^: the four clinicians that stated that ILD treatment was not known were not followed with the questions in b; only those that stated that a given ILD drug was being used in their patients in a were given the follow-up question for the specific drug in b.

Regarding ILD management, 87% of clinicians reported using immunosuppressant and antifibrotic medications, respectively, in any number of their PH-ILD patients to control underlying ILD (n=51 responses; four clinicians answered “not known”; figure 2a). Around half (median 50%, IQR 30–70%) of patients were prescribed antifibrotics such as nintedanib and pirfenidone (n=48 responses) and a median 23% (IQR 15–50%) received immunosuppressants (n=48 responses; figure 2b).

**FIGURE 3:**
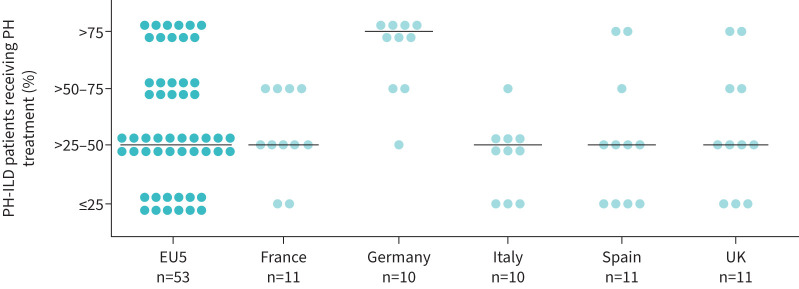
Off-label PAH treatment frequency in PH-ILD patients managed by clinicians (n=53^#^). Each circle represents one clinician's answer; bar represents the mean value. PAH: group 1 pulmonary hypertension; PH-ILD: pulmonary hypertension associated with interstitial lung disease; EU5: European five countries (France, Germany, Italy, Spain and the UK). ^#^: two clinicians did not answer this question.

To manage the PH component of PH-ILD, clinicians prescribed off-label PAH treatments to a median 50% (IQR 30–70%) of their PH-ILD patients (n=53; two respondents did not provide an overall proportion). Off-label PAH treatment frequency in PH-ILD patients varied amongst the countries, with Italian, Spanish, French, UK and German clinicians declaring that a median 40% (IQR 26–50%), 30% (IQR 15–55%), 35% (IQR 30–63%), 50% (IQR 25–75%) and 100% (IQR 73–100%) of their PH-ILD patients received off-label PAH therapy, respectively (figure 3).

Regarding the underlying rationale in treating PH in PH-ILD, clinicians cited the lack of effective PH treatments (33%) and that PH severity had not reached a sufficient level to warrant treatment (31%) as reasons for not addressing the PH component. Additional factors cited were the risk of adverse events (18%) and the absence of clear treatment guidelines (16%) (supplementary table S2). Clinicians agreed that PH severity (91%), ILD severity (75%) and right ventricular function (65%) were of high relevance when considering PH treatment.

**FIGURE 4:**
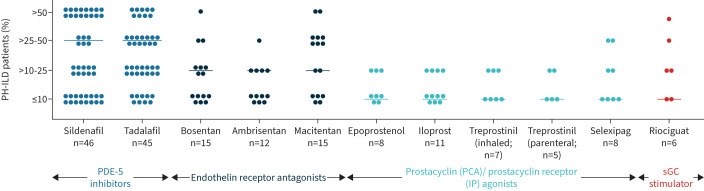
Off-label PAH monotherapy frequency in PH-ILD patients managed by clinicians (n=55). Each circle represents one clinician's answer; bar represents the mean value. PDE-5: phosphodiesterase-5; PAH: group 1 pulmonary hypertension; PH-ILD: pulmonary hypertension associated with interstitial lung disease; sGC: soluble guanyl cyclase.

Most PH-ILD patients were prescribed supplemental oxygen therapy (median 80%, IQR 50–90%). Nearly all clinicians (95%) reported using off-label PAH monotherapy (in any line), including PDE-5i (87%), ERA (35%), PCA (25%) or sGC stimulators (11%) (figure 4). 55% of clinicians (n=30) reported using dual-therapy in their PH-ILD patients. The majority prescribed a combination of PDE-5i and ERA (76%). Nearly 80% (78%) indicated having used PDE-5i as a first-line PH treatment choice. A minority prescribed ERA (9%) and PCA (7%) as first-line therapies. Upon non-response to a first-line PDE-5i, clinicians prescribed as second-line either ERA (35%), PCA (15%,), another PDE-5i (15%) or a sGC stimulator (7%). Over a third of clinicians (35%) ceased PH treatment if their patients did not respond to first-line PDE-5i treatment.

### Management and follow-up

To assess the impact of PH in ILD patients, clinicians were surveyed regarding the frequency of patient follow-ups, particularly among those listed for lung transplantation and the tests and procedures routinely employed for PH-ILD patients. A median 20% (IQR 8–55%), 50% (IQR 28–65%) and 10% (IQR 0–23%) of PH-ILD patients were consulted between 1–2, 3–4 and 5–6 times per year, respectively. For PH-ILD patients on a lung transplant list, a median 0% (IQR 0–30%), 20% (IQR 0–60%) and 5% (IQR 0–50%) were seen between 1–2, 3–4 and 5–6 times per year, respectively. The remaining patients were followed up >6 times per year.

To monitor PH disease progression, most clinicians assessed oxygen saturation (75%), biomarker levels (any type) (42%) and exercise capacity (6-min walk distance; 31%) for their PH-ILD patients at every follow-up visit. Other clinical investigations such as echocardiogram (45%) and PFT (36%) were performed every 6 months. RHC was performed annually by most clinicians (60%). Regarding captured outcomes, nearly three-quarters of clinicians (73%) reported regularly capturing both pulmonary function and oxygen saturation levels (table 2). Regarding continuation or discontinuation rationale for PH treatment, most clinicians continued PH treatments if exercise capacity (85%), haemodynamic parameters (71%) and QoL (67%) measures were maintained or improved. All discontinued PH treatment in their PH-ILD patients upon adverse event (desaturation or tolerability) occurrence, with other reported discontinuation factors being comorbidity development (51% of clinicians), PH progression (38%) and ILD progression (33%).

**TABLE 2:** Regularly captured clinical outcomes in PH-ILD patients managed by clinicians (n=55)

**Oxygen saturation levels**	73 (40)
**Pulmonary function (*i.e.*, FVC)**	73 (40)
**Exercise capacity (*e.g.*, 6MWT, CPET)**	71 (39)
**Echocardiographic measurements**	67 (37)
**Serological biomarkers (BNP, NT-ProBNP)**	65 (36)
**Diffusing capacity of the lung for carbon monoxide**	62 (34)
**Cardiopulmonary hospitalisation**	53 (29)
**Episodes of acute exacerbations of ILD**	45 (25)
**Haemodynamic parameters (*i.e.*, mPAP, PVR)**	27 (15)
**Number of lung disease exacerbations**	27 (15)
**Quality of life (patient-reported outcomes)**	16 (9^#^)

Data are presented as n (%). PH-ILD: pulmonary hypertension associated with interstitial lung disease; FVC: forced vital capacity; 6MWT: 6-min walk test; CPET: cardiopulmonary exercise test; BNP: brain natriuretic peptide; NT-ProBNP: N-terminal pro-brain natriuretic peptide; ILD: interstitial lung disease; mPAP: mean pulmonary arterial pressure; PVR: pulmonary vascular resistance. ^#^: of the nine clinicians that selected quality of life as a regularly captured outcome, six were from the UK.

**FIGURE 5:**
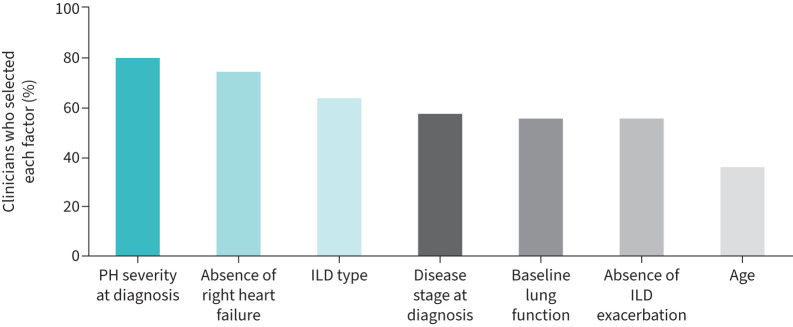
Clinician-selected prognostic factors at diagnosis of pulmonary hypertension (PH) for a positive response to off-label PAH therapy in PH-ILD patients (n=55). ILD: interstitial lung disease; PAH: group 1 pulmonary hypertension.

The most relevant reported prognostic factors predicting a positive outcome to PH therapy were PH severity at diagnosis (82% of clinicians) and absence of right heart failure (76%) (figure 5). When asked about the reasons for hospitalisation, clinicians reported a median 30% (IQR 20–40%) of PH-ILD patients were hospitalised due to right heart failure. Other reasons consisted of acute ILD exacerbation, symptom burden and comorbidity severity, which were reported as the reason for a median 30% (IQR 10–40%), 20% (IQR 10–30%) and 20% (IQR 10–20%) of PH-ILD patients, respectively. In a hospitalisation frequency question fielded after survey completion (n=31 clinicians answered), clinicians reported that median yearly hospitalisation frequency per PH-ILD patient was 2.0 (IQR 1.3–2.9) for inpatient admissions and 1.5 (IQR 1–2) for emergency visits.

**FIGURE 6:**
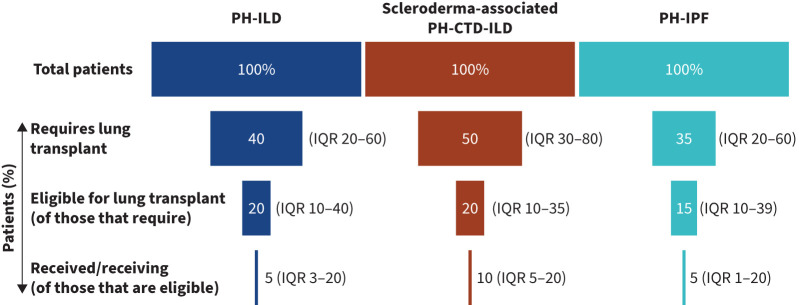
Median clinician-reported lung transplantation rates for their PH-ILD patients (n=55). PH-CTD-ILD: pulmonary hypertension associated with connective tissue disease-based interstitial lung disease; PH-ILD: pulmonary hypertension associated with interstitial lung disease; PH-IPF: pulmonary hypertension associated with idiopathic pulmonary fibrosis.

When asked what proportion of their patients required lung transplantation, median clinician-reported values were 40% (IQR 20–60%), 50% (IQR 30–80%) and 35% (IQR 20–60%) for their PH-ILD, PH-IPF and scleroderma-associated PH-ILD patients, respectively. Of those, the median proportion of PH-ILD, PH-IPF and scleroderma-associated PH-ILD patients who were eligible for lung transplant was 20% (IQR 10–40%), 20% (IQR 10–35%) and 15% (10–39%), respectively. Finally, of those, the median proportion of PH-ILD, PH-IPF and scleroderma-associated PH-ILD patients who received a lung transplantation was 5% (IQR 3–20%), 10% (IQR 5–20%) and 5% (IQR 1–20%), respectively (figure 6).

Clinicians suggested greater collaboration between multidisciplinary teams (25%), more evidence-based guidelines (25%) and more efficacious PH treatments (24%) to improve the management of PH-ILD (supplementary table S3).

## Discussion

This represents the first European real-world survey delving into the management of PH-ILD patients. Our research has unveiled distinctive insights into European clinicians' perspectives on PH-ILD patients, underscoring the considerable unmet needs in the domain of PH-ILD management. This includes the demand for robust screening and diagnostic protocols, as well as effective, officially approved therapies across Europe. Despite observed country-specific differences, only around half of ILD patients in Europe suspected to have PH are undergoing diagnostic RHC. Once diagnosed, only around half are receiving treatment for their PH component. PH-ILD patients are on average ≥60 years old, with severe ILD. Nearly 70% of patients have been diagnosed with their ILD ≥2 years ago. This severity may explain the frequent hospital visits, which were ∼3.5 per year per patient based on median values. With this severity, PH-ILD patients are often followed up on a quarterly basis, but according to clinician perception, outcomes in PH-ILD are heavily dependent upon PH severity, which lacks effective management tools.

Overall, participating clinicians felt that PH is not being screened with sufficient regularity, and that the currently available off-label treatments fall short in their efficacy, especially in cases of mild-to-moderate PH. Indeed, severity of PH is often perceived as not severe enough to justify treatment, tying in with clinician comments on the need for awareness and education, which may help to oppose this perception and increase treatment frequency.

While there is a limited literature on the diagnosis of PH in ILD, a Delphi panel in the US reported clinician consensus on PH suspicion [10]. These clinicians had the greatest consensus on using right ventricle enlargement and increased pulmonary artery/aorta ratio as markers to suspect PH in ILD patients. Physicians in our study most commonly selected the parameters of PFT (low *D*_LCO_ and elevated % FVC/% *D*_LCO_ ratio) and mPAP as crucial for suspecting PH in ILD, neither of which were mentioned in the Delphi report. However, our third most common choice was “imaging findings” (*e.g.*, ventricle enlargement, pulmonary artery/aorta ratio), showing that our PH suspicion findings are in line with other investigations. This focus on PFTs is likely due to the high proportion of pulmonologists enrolled in our questionnaire, as when we only analysed the signs chosen by cardiologists, mPAP and imaging findings were more important. With the lack of an equivalent study in Europe, clinician comments reinforce the notion that efforts should be focused on education, collaboration of experts and generating more evidence on PH-ILD and its subtypes to support more evidence-based guidelines. This will lead to better management of PH-ILD, even without approved therapies.

Our treatment findings also align with adjacent literature. Before the FDA approval of treprostinil in PH-ILD, a US health claims study for group 3 PH patients found that around 30% of PH-ILD patients were receiving PDE-5i, and 23% were receiving ERA [12]. This is in line with our mean ranges of off-label PAH therapy usage for PDE-5i (25–50%) and ERA (10–25%). Additionally, a German PH registry reported off-label PAH treatment usage, and for their group 3 PH cohort (52% were PH-ILD), 22% received no specific PH therapy [13], in line with our Germany-specific PH treatment frequency of ∼80%.

The study's limitations arise from its retrospective design, which can introduce recall bias, and its relatively small sample size consisting of 55 clinicians (43 pulmonologists, nine cardiologists and three rheumatologists) that mainly practised at academic or research centres. As a result, the country-specific findings may not accurately reflect their respective populations or the opinions of general pulmonologists. Moreover, the survey encompassed clinicians who either personally managed patient cohorts or co-managed them with other clinicians, potentially leading to significant variations in cohort sizes. Furthermore, we could not verify the data source the clinicians used for their PH-ILD records. To substantiate the findings of this survey, future research could delve into pulmonology centre registry data to collect statistics on therapy usage based on prescription data.

Overall, we have shown that practising clinicians are often not diagnosing and treating PH-ILD due to the lack of frequent screening and effective therapies. With the availability of targeted PH-ILD therapies, clinicians may have a clearer purpose in diagnosing and treating PH-ILD patients, eventually leading to the implementation of diagnostic and treatment guidelines and improved outcomes for this severe patient group. A key marker of success for PH-ILD management in the future will be an effective multidisciplinary approach and improved education for this complex disease, which a significant proportion of practising clinicians agreed on.

## Supplementary material

10.1183/23120541.00039-2024.Supp1 **Please note:** supplementary material is not edited by the Editorial Office, and is uploaded as it has been supplied by the author.
Supplementary material 00039-2024.SUPPLEMENT
