# Supplementary material for: Unmet need in pulmonary hypertension-associated interstitial lung disease (PH-ILD): a clinician survey of real-world management of PH-ILD in Europe
Source: ERJ Open Res. 2024 Jul 8;10(4):00039-2024. doi: 10.1183/23120541.00039-2024 (PMC11228598; doi:10.1183/23120541.00039-2024)
Supplement: Supplementary file 1 [file 00039-2024.SUPPLEMENT.pdf]

Supplementary item 1. Full questionnaire

1. In which country do you practice?

☐France
☐Germany
☐Italy
☐Spain
☐United Kingdom
☐Other (please specify):

2. What is your clinical specialisation?

☐Cardiology
☐Pulmonology/Pneumonology/Respiratory Medicine
☐Rheumatology
☐Other (please specify):

3. Of the following, which disease(s) do you manage (diagnose/treatment/follow-up)?

☐PH
☐ILD
☐Both
☐None of the above

4. Please state your years of experience in caring for PH/ILD/PH-ILD patients:

☐0-2 years
☐3-5 years
☐6-10 years
☐10+ years

5. What best describes your main practice setting?

☐Public general hospital
☐Academic/university/research health centre
☐Public specialised pulmonary care centre
☐Private general hospital
☐Private specialist pulmonary centre
☐Other (please specify):

6. Does your centre carry out the following procedures?

☐Echocardiography
☐Right heart catheterisation (RHC)

☐Lung transplant
☐None of the above

7. Do you participate in any PH (any WHO functional class, 1-4) / ILD (e.g., IPF and CTD) /PH-ILD registries?

☐No
☐Yes (Please specify name of registries):

8. Are you a clinical investigator on any ongoing/completed PH (any WHO functional class, 1-4) / ILD (e.g., IPF and CTD) or PH-ILD clinical trials?

☐No
☐Yes (please elaborate):

For the remainder of the questionnaire, the term PH-ILD patient, will refer to those with a confirmed interstitial lung disease and PH confirmed through right heart catheterisation (RHC) or those with a high probability of PH from echocardiography

9. What is the approximate number of patients managed at your centre in the last 12 months? If not known, please state in comments

PH patients	
ILD patients	
PH-ILD patients	

Comments:

10. What is the approximate number of patients you personally manage/co-manage annually? If not known, please state in the comments

PH patients	
ILD patients	
PH-ILD patients	

Comments:

PH-ILD patient characteristics

For the following questions, please respond based on your experience in managing PH-ILD patients (confirmed interstitial lung disease and PH confirmed through right heart catheterisation (RHC) or those with a high probability of PH from echocardiography)

11. Approximately, what is the gender distribution of your PH-ILD patients?

Male % of PH-ILD patients

Female % of PH-ILD patients

Total: % of PH-ILD patients

12. Approximately, what is the age distribution of your PH-ILD patients?

≥60 years % of PH-ILD patients

<60 years % of PH-ILD patients

Total: % of PH-ILD patients

13. Approximately, what is the percentage distribution of your PH-ILD patients based on time since ILD diagnosis?

≥2 years diagnosed with ILD % of PH-ILD patients

<2 years diagnosed with ILD % of PH-ILD patients

Total: % of PH-ILD patients

Please state if not known due to specialisation:

14. Approximately, what is the percentage distribution of your PH-ILD patients based on time since PH diagnosis?

< 6 months % of PH-ILD patients

6 months – 1 year % of PH-ILD patients

>1 year % of PH-ILD patients

Total: % of PH-ILD patients

Please state if not known due to specialisation:

15. Approximately, what is the distribution of your PH-ILD patients based on ILD type/cause?

Idiopathic pulmonary fibrosis (IPF)

	% of PH-ILD patients
Non-specific interstitial pneumonia (NSIP)	% of PH-ILD patients
Scleroderma associated connective tissue disease (CTD-ILD)	% of PH-ILD patients
Non-scleroderma associated CTD-ILD	% of PH-ILD patients
Sarcoidosis	% of PH-ILD patients
Combined pulmonary fibrosis and emphysema (CPFE)	% of PH-ILD patients
Other (Please specify in comments)	% of PH-ILD patients
**Total:**	% of PH-ILD patients

Comments:

16. Approximately, what is the distribution of your PH-ILD patient based on WHO functional class at diagnosis of PH?

WHO functional class 1-2	% of PH-ILD patients
WHO functional class 3-4	% of PH-ILD patients
**Total:**	% of PH-ILD patients

State if not known due to specialisation:

17. Approximately, what is the distribution of your PH-ILD patient based on predicted forced vital capacity (FVC) at diagnosis of PH?

<50% forced vital capacity	% of PH-ILD patients
50-70% forced vital capacity	% of PH-ILD patients
>70% forced vital capacity	% of PH-ILD patients
**Total:**	% of PH-ILD patients

State if not known due to specialisation:

18. On average, with what frequency are the following symptoms and signs seen in your PH-ILD patients? [The choices are: High (observed in >50% of patients), Medium (Observed in 25-50% of patients) and Low (observed in <25% of patients)]

	Frequency of symptom or signs
Chest pain	☐
Cough	☐
Dizziness	☐
Dyspnoea	☐
Fatigue	☐
Headache	☐
Palpitations	☐
Sleep disturbances	☐
Swelling	☐
Syncope	☐

Other (Please specify):

19. On average, with what frequency are the following comorbidities seen in your PH-ILD patients? [The choices are: High (observed in >50% of patients), Medium (Observed in 25-50% of patients) and Low (observed in <25% of patients)]

	Frequency of comorbidity
Arthritis	☐
Atrial fibrillation	☐
COPD	☐
Coronary artery disease	☐
Diabetes	☐
Hypertension	☐
Left heart disease	☐
Obesity	☐
Pulmonary emboli	☐
Sleep apnoea	☐

Other (Please specify):

Diagnostic pathway

In this section we would like to characterise the methods/rationale that were used to diagnose PH in ILD patients

20. What guidelines / recommendations do you use to diagnose PH in ILD patients?

☐6th World Symposium on Pulmonary Hypertension (WSPH)
☐European Society of Cardiology (ESC; 2015)
☐Not Known (PH diagnosis is not my expertise)
☐Other (eg: local clinical guidelines):

21. What are the key clinical, functional and/or imaging parameters that you consider in ILD patients to suspect PH? (Please select up to 5 options)

☐Abnormal pulmonary function tests (low DLCO, elevated %FVC / %DLCO ratio)
☐Decreased exercise capacity (6MWT, CPET)
☐Dyspnoea out of proportion
☐ECG signs
☐Elevated biomarker levels (eg: BNP, NT-proBNP)
☐Exercise desaturation
☐High pulmonary arterial pressure
☐History of pulmonary embolism
☐Imaging findings (enlarged PA, PA / aorta diameter ratio >1 on CT / dilated RV)
☐Peak VO2/kg/min
☐Resting desaturation
☐Signs of right heart failure
☐Other (please specify):

22. At your centre, how are ILD patients referred for right heart catheterisation (RHC, to confirm PH diagnosis)?

☐Patients are referred to a PH specialist at your centre
☐Patients are referred to other PH specialist centres
☐Other (please specify):

23. Approximately, what proportion of ILD patients with suspected PH are referred for the following procedures?

Echocardiography only — % of PH-ILD patients

Echocardiography followed by right heart catheterisation (RHC) — % of PH-ILD patients

Right heart catheterisation (RHC) alone — % of PH-ILD patients

Other

% of PH-ILD patients

Total:

% of PH-ILD patients

If another procedure was used, please specify what it was:

24. Based on your experience, please elaborate in which cases you would or would not carry out a right heart catheterisation (RHC) in ILD patients

25. Given a suspicion of PH, approximately how long does it take to confirm a PH diagnosis through the use of right heart catheterisation (RHC)?

☐<2 weeks
☐2-4 weeks
☐>4 weeks

26. What is the approximate relative distribution of mPAP outcomes at diagnosis of PH for your PH-ILD patients? *

21-24mmHg % of PH-ILD patients

25-34mmHg % of PH-ILD patients

35mmHg+ % of PH-ILD patients

Total: % of PH-ILD patients

Comments:

27. In your opinion, what are the key areas and actions needed to improve diagnosis of PH-ILD patients?

Treatment pathway

In this section we would like to understand how PH-ILD patients are treated at your centre

28. How are your PH-ILD patients currently treated for their underlying ILD? Please select all therapies that apply:

☐Antifibrotic (nintedanib, pirfenidone)
☐Anti-inflammatory (prednisone, methylprednisolone)
☐Immunosuppressants (azathioprine, methotrexate, mycophenolate)
☐Rituximab

☐TNF inhibitors
☐Not known (ILD treatment outside of my specialisation)

Others (please specify names and if known, the % of your PH-ILD patients that are receiving):

29. Approximately, what % of your PH-ILD patients are receiving antifibrotics (nintedanib, pirfenidone) to treat their underlying ILD?

30. Approximately, what % of your PH-ILD patients are receiving anti-inflammatory therapy (prednisone, methylprednisolone) to treat their underlying ILD?

31. Approximately, what % of your PH-ILD patients are receiving immunosuppressant therapy (azathioprine, methotrexate, mycophenolate) to treat their underlying ILD?

32. Approximately, what % of your PH-ILD patients are receiving rituximab to treat their underlying ILD?

33. Approximately, what % of your PH-ILD patients are receiving TNF inhibitor therapy to treat their underlying ILD?

34. Do all of your PH-ILD patients receive specific treatment for PH?

☐Yes
☐No (% of your PH-ILD patients not receiving treatment for PH):

35. What are the reasons for not treating PH in these PH-ILD patients?

36. According to your experience in managing PH-ILD patients, what class of drugs are used as a monotherapy to manage PH in these patients?

☐Endothelin receptor antagonists (ERA)
☐Phosphoiesterase-5-inhibitors (PDE5i)
☐Prostacyclin analogues (PCA) / Prostacyclin receptor (IP) agonists
☐Soluble guanylate cyclase (sGC) stimulators
☐Other (please specify):

37. According to your experience, which phosphodiesterase-5 inhibitors (PDE-5i) are used as monotherapy to manage PH in your PH-ILD patients? If applicable, please select the proportion of your PH-ILD patients that receive this treatment.

	Not used	<10% of PH-ILD patients	10-25% of PH-ILD patients	25-50% of PH-ILD patients	>50% of PH-ILD patients
Sildenafil	☐	☐	☐	☐	☐
Tadalafil	☐	☐	☐	☐	☐
Other	☐	☐	☐	☐	☐

If others were selected, please specify the drug:

38. According to your experience, which prostacyclin analogues (PCA) / Prostacyclin receptor (IP) agonists are used as monotherapy to manage PH in your PH-ILD patients? If applicable, please select the proportion of your PH-ILD patients that receive this treatment.

	Not used	<10% of PH-ILD patients	10-25% of PH-ILD patients	25-50% of PH-ILD patients	>50% of PH-ILD patients
Epoprostenol	☐	☐	☐	☐	☐
Iloprost (Inhaled)	☐	☐	☐	☐	☐
Selexipag	☐	☐	☐	☐	☐
Treprostinil (inhaled)	☐	☐	☐	☐	☐
Treprostinil (parental)	☐	☐	☐	☐	☐
Other	☐	☐	☐	☐	☐

If others were selected, please specify the drug:

39. According to your experience, which endothelin receptor antagonists (ERAs) are used as monotherapy to manage PH in your PH-ILD patients? If applicable, please select the proportion of your PH-ILD patients that receive this treatment.

	Not used	<10% of PH-ILD patients	10-25% of PH-ILD patients	25-50% of PH-ILD patients	>50% of PH-ILD patients
Ambrisentan	☐	☐	☐	☐	☐
Bosentan	☐	☐	☐	☐	☐
Macitentan	☐	☐	☐	☐	☐
Other	☐	☐	☐	☐	☐

If others were selected, please specify the drug:

40. According to your experience, which soluble guanylate cyclase (sGC) stimulators are used as monotherapy to manage PH in your PH-ILD patients? If applicable, please select the proportion of your PH-ILD patients that receive this treatment.

	Not used	<10% of PH-ILD patients	10-25% of PH-ILD patients	25-50% of PH-ILD patients	>50% of PH-ILD patients
Riociguat	☐	☐	☐	☐	☐
Other	☐	☐	☐	☐	☐

If others were selected, please specify the drug:

41. According to your experience, are combinational therapies used to manage PH in your PH-ILD patients?

☐Yes
☐No

42. According to your experience/opinion what are the most relevant combinational (dual) therapies that could be used to manage PH in PH-ILD patients? Please select the combinations that apply from the table below:

	Sildenafil	Tadalafil	Bosentan	Macitentan	Ambrisentan	Riociguat	Treprostinil (Inhaled)	Treprostinil (Parenteral)	Epoprostenol	Iloprost
Sildenafil	☐	☐	☐	☐	☐	☐	☐	☐	☐	☐
Tadalafil	☐	☐	☐	☐	☐	☐	☐	☐	☐	☐
Bosentan	☐	☐	☐	☐	☐	☐	☐	☐	☐	☐
Macitentan	☐	☐	☐	☐	☐	☐	☐	☐	☐	☐
Ambrisentan	☐	☐	☐	☐	☐	☐	☐	☐	☐	☐
Riociguat	☐	☐	☐	☐	☐	☐	☐	☐	☐	☐
Treprostinil (Inhaled)	☐	☐	☐	☐	☐	☐	☐	☐	☐	☐
Treprostinil (Parenteral)	☐	☐	☐	☐	☐	☐	☐	☐	☐	☐
Epoprostenol	☐	☐	☐	☐	☐	☐	☐	☐	☐	☐
Iloprost	☐	☐	☐	☐	☐	☐	☐	☐	☐	☐

43. When are the above combinational therapies used to manage PH in PH-ILD patients?

44. Based on your experience, what proportion (%) of your PH-ILD patients are receiving supplemental oxygen therapy?

45. Based on your experience, are triple therapies also used to treat PH in your PH-ILD patients? If so, please elaborate on what combinations are used and when:

46. According to your experience, what are the primary PH treatment choices for PH-ILD patients? If the patient does not respond, what are the secondary treatment options?

Please indicate an initial therapy and then a potential alternative therapy, if no other therapy would be given after non-response, leave "secondary therapy" blank

	First choice (1L)	Secondary therapy (2L)
Option 1		
Option 2		

	First choice (1L)	Secondary therapy (2L)
Option 3		

Comments:

47. Based on your experience, how relevant are the following factors when considering treatments for PH in PH-ILD patients?

	Low relevance	Med relevance	High relevance
ILD condition	☐	☐	☐
On lung transplant waiting list	☐	☐	☐
Presence of comorbidities	☐	☐	☐
QoL of patient	☐	☐	☐
Severity of ILD	☐	☐	☐
Severity of PH	☐	☐	☐
RV function	☐	☐	☐

Others (Please specify and elaborate on the other factor/s):

48. Based on your experience, please evaluate the relative importance of the following ILD conditions for PH treatment choice (in PH-ILD patients):

	Low relevance	Medium relevance	High relevance
CPFE	☐	☐	☐
CTD (non-scleroderma-associated)	☐	☐	☐
CTD (scleroderma-associated)	☐	☐	☐
IPF	☐	☐	☐
NSIP	☐	☐	☐
Sarcoidosis	☐	☐	☐

49. Based on your experience, please evaluate the relative importance of the following mPAP ranges for PH treatment choice (in PH-ILD patients):

	Low relevance	Medium relevance	High relevance
20-25mmHg	☐	☐	☐
25-35mmHg	☐	☐	☐
>35mmHg	☐	☐	☐

50. Based on your experience, please evaluate the relative importance of the following comorbidities for PH treatment choice (in PH-ILD patients):

	Low relevance	Medium relevance	High relevance
COPD	☐	☐	☐
Heart failure	☐	☐	☐
Obesity	☐	☐	☐
Pulmonary emboli	☐	☐	☐

Others (Please specify):

51. Based on your experience, please elaborate on how a listing on a lung transplant waiting list would impact PH treatment choice in PH-ILD patients?

52. Based on your experience, please elaborate on how patient's QoL would determine PH treatment choice in PH-ILD patients?

53. Based on your experience, please evaluate the relative importance of the following ILD severity parameters for PH treatment choice (in PH-ILD patients):

	Low relevance	Medium relevance	High relevance
Mild ILD	☐	☐	☐
Moderate ILD	☐	☐	☐
Severe ILD	☐	☐	☐

Comments:

Based on your experience, please elaborate on how patient RV function would determine PH treatment choice in PH-ILD patients?

54. In your opinion, what are the key areas and actions needed to improve the treatment pathway of PH-ILD patients?

Disease management and follow-up

In this section we would like to understand the methods used at your clinic for the follow up for PH-ILD patients

55. How frequently do you see your PH-ILD patients in a year?

1-2 times per year	______ % of PH-ILD patients
3-4 times per year	______ % of PH-ILD patients
5-6 times per year	______ % of PH-ILD patients
7-8 times per year	______ % of PH-ILD patients
8-9 times per year	______ % of PH-ILD patients
10+ times per year	______ % of PH-ILD patients
**Total:**	______ % of PH-ILD patients

56. How frequently do you see your PH-ILD patients on a lung transplant waiting list in a year?

1-2 times per year	______ % of PH-ILD patients
3-4 times per year	______ % of PH-ILD patients
5-6 times per year	______ % of PH-ILD patients
7-8 times per year	______ % of PH-ILD patients
8-9 times per year	______ % of PH-ILD patients
10+ times per year	______ % of PH-ILD patients
**Total:**	______ % of PH-ILD patients

57. What tools are used to monitor PH disease progression in your PH-ILD patients?

	Not used	At every follow up	Once a month	Once every 3 months	Once every 6 months	Annually
Biomarker levels						
Diffusing Capacity Of The Lungs For Carbon Monoxide (DLCO)	☒	☐	☐	☐	☐	☐
Echocardiography	☐	☐	☐	☐	☐	☐
Exercise testing (6MWD)	☐	☐	☐	☐	☐	☐
Haemodynamic parameters (e.g., mPAP, PVR)	☐	☐	☐	☐	☐	☐
High resolution computed tomography	☐	☐	☐	☐	☐	☐
Oxygen saturation	☐	☐	☐	☐	☐	☐
Pulmonary Function (e.g., FVC)	☐	☐	☐	☐	☐	☐

☐ Others (please specify technique and state frequency of administration as well):

58. In your opinion, what are the key areas and actions needed to improve the disease management and follow-up in PH-ILD patients?

59. Which of the following outcomes do you regularly capture/record in your PH-ILD patients? (Please select up to 6 options)

☐ Cardiopulmonary hospitalisation
☐ Diffusing capacity for carbon monoxide
☐ Echocardiographic measurements
☐ Episodes of acute exacerbations of ILD
☐ Exercise capacity (e.g., 6MWT, CPET)

☐ Haemodynamic parameters (i.e., mPAP, PVR)
☐ Number of lung disease exacerbations
☐ Oxygen saturation levels
☐ Pulmonary function (i.e., FVC)
☐ Quality of life (Patient Reported Outcomes)
☐ Serological Biomarkers (BNP, NT-ProBNP)
☐ Other (please specify):

60. Based on your experience, which are the key prognostic factors that could predict a positive outcome for PH treatment in PH-ILD patients? (please select up to 5 options)

☐ Absence of exacerbation of ILD
☐ Absence of right heart failure
☐ Age
☐ Baseline lung function
☐ Disease stage at diagnosis (WHO function classification)
☐ ILD type
☐ PH severity at diagnosis
☐ Other (please specify):

61. Based on your experience, please elaborate on how age is a key prognostic factor for positive response to PH treatment in PH-ILD patients

62. Based on your experience, please elaborate on how disease stage is a key prognostic factor for positive response to PH treatment in PH-ILD patients

63. Based on your experience, please elaborate on how ILD type is a key prognostic factor for positive response to PH treatment in PH-ILD patients

64. Based on your experience, please elaborate on how baseline lung function is a key prognostic factor for positive response to PH treatment in PH-ILD patients

65. Based on your experience, please elaborate on how PH severity at diagnosis is a key prognostic factor for positive response to PH treatment in PH-ILD patients

66. Based on your experience, please elaborate on how exacerbation of ILD is a key prognostic factor for positive response to PH treatment in PH-ILD patients

67. Based on your experience, please elaborate on how presence/absence of right heart failure is a key prognostic factor for positive response to PH treatment in PH-ILD patients

68. Based on your experience, please elaborate on how this "other" key factor predicts for a positive response to PH therapy in PH-ILD patients

69. According to your experience, which key outcomes/factors do you consider for PH treatment continuation in PH-ILD patients? (please select up to 5 options)

☐Maintenance/improvement of haemodynamic parameters
☐Maintenance/improvement of pulmonary parameters
☐Maintenance/improvement of exercise capacity (6MWT, CPET)
☐Maintenance/improvement in serological markers
☐Maintenance/improvement of patient's quality of life
☐Non-eligibility to lung transplant
☐Other (please specify):

70. According to your experience, which key factors are considered to discontinue PAH treatment in PH-ILD patients?

☐ Adverse response (eg: desaturation, tolerability, dyspnea)
☐ Comorbidity development
☐ ILD progression
☐ PH progression
☐ Other (please specify):

71. According to your experience, approximately what proportion of PH-ILD patients are hospitalised due to the following reasons?

Acute exacerbation of ILD	% of PH-ILD patients
Comorbidity severity	% of PH-ILD patients
Right heart failure	% of PH-ILD patients
Severe symptom burden	% of PH-ILD patients
Other	% of PH-ILD patients
**Total:**	% of PH-ILD patients

If "other" was selected, please specify the reason:

72. According to your knowledge and experience, what is the life expectancy (years) after PH diagnosis for the following patient types?

PH-ILD patients

PH-IPF patients

Scleroderma associated PH-CTD-ILD

73. Approximately, what proportion of your PH-ILD and PH-IPF patients require lung transplant? Of these, what proportion of patients are eligible and what proportion of patients actually receive the transplant?

	% of patients requiring a lung transplant	% that are eligible for transplant (of those that require transplant)	% that received / are receiving transplant (of those that are eligible for transplant)
PH-ILD			
PH-IPF			
Scleroderma associated PH-CTD-ILD			

Supplementary table S1. Clinicians' perspectives towards the diagnosis of PH-ILD (free text answers)

**Question**	**Proportion of responses** (%, n)
**Q24**. Based on your experience, please elaborate in which cases you would or would not carry out a right heart catheterisation (RHC) in ILD patients	
As a treatment/transplant candidate	24% (13)
Moderate/severe PH with mild/moderate ILD	16% (9)
If there is a high probability from echocardiogram	16% (9)
Would not, with severe ILD	15% (8)
In those with disproportionate PH	13% (7)
Would not, with those who wouldn't tolerate procedure (frail, comorbidity burden)	13% (7)
Sarcoidosis/scleroderma patients with suspected PH	11% (6)
Those with severe right heart failure	11% (6)
**Q27**. In your opinion, what are the key areas and actions needed to improve diagnosis of PH-ILD patients?	
More frequent screening for PH (using echocardiogram)	35% (19)
Education and awareness of PH amongst clinicians	24% (13)
Better screening methods (biomarkers, radiological tests, improved	15% (8)

echocardiogram)	
More frequent / quicker RHC	13% (7)
More treatments of greater efficacy	11% (6)

Abbreviations: ILD, Interstitial lung disease; PH, pulmonary hypertension; PH-ILD, pulmonary hypertension associated with interstitial lung disease; RHC, right heart catheterisation

Supplementary table S2. Clinicians' perspectives towards the treatment of PH in ILD patients (free text answers)

Question	Proportion of responses (%, n)
**Q35.** What are the reasons for not treating PH in these PH-ILD patients?	
Lack of efficacious PH treatments	33% (18)
PH severity not reaching the treatment threshold (mild to moderate PH)	31% (17)
Risk of adverse events	18% (10)
Lack of treatment guidelines	16% (9)
Severe ILD	7% (4)
ILD aetiology	4% (2)
Non-vascular phenotype	2% (1)
Left heart disease	2% (1)

Abbreviations: ILD, interstitial lung disease PH, pulmonary hypertension; PH-ILD, pulmonary hypertension associated with interstitial lung disease

Supplementary table S3. Clinicians' perspectives towards the follow-up of PH-ILD patients (free text answers)

Question	Proportion of responses (%, n)
**Q58.** In your opinion, what are the key areas and actions needed to improve the disease management and follow-up in PH-ILD patients?	
Multidisciplinary collaborations and need of more disease centres	25% (14)
Evidence-based guidelines	25% (14)
Efficacious PH treatments	24% (13)
Better disease monitoring tools (imaging techniques)	13% (7)
Early PH diagnosis	7% (4)
Better disease awareness	4% (2)
Better access to disease monitoring tools and remote consultation	4% (2)
Prevention of exacerbation episodes and comorbidities development	2% (1)

Abbreviations: PH-ILD, pulmonary hypertension associated with interstitial lung disease; PH, pulmonary hypertension
